# Exposure to Iron Oxide Nanoparticles Coated with Phospholipid-Based Polymeric Micelles Induces Biochemical and Histopathological Pulmonary Changes in Mice

**DOI:** 10.3390/ijms161226173

**Published:** 2015-12-10

**Authors:** Mihaela Radu (Balas), Ioana Mihaela Din (Popescu), Anca Hermenean, Otilia Ludmila Cinteză, Radu Burlacu, Aurel Ardelean, Anca Dinischiotu

**Affiliations:** 1 Department of Biochemistry and Molecular Biology, Faculty of Biology, University of Bucharest, 91-95 Splaiul Independentei, Bucharest 050095, Romania; mihaela.radu@bio.unibuc.ro (M.R.B.); ioana.mihaela.din@gmail.com (I.M.D.P.); 2 Department of Experimental and Applied Biology, Institute of Life Sciences, Vasile Goldis Western University of Arad, 86 Rebreanu, Arad 310414, Romania; biologie@uvvg.ro; 3 Department of Histology, Faculty of Medicine, Vasile Goldis Western University of Arad, 1 Feleacului, Arad 310396, Romania; 4 Department of Physical Chemistry, Faculty of Chemistry, University of Bucharest, 4-12 Regina Elisabeta Blvd, Bucharest 030018, Romania; ocinteza@gw-chimie.math.unibuc.ro; 5 Department of Mathematics, University of Agriculture Sciences and Veterinary Medicine, 59 Marasti, Bucharest 011464, Romania; radu.burlacu@fifim.ro

**Keywords:** phospholipid micelles-coated iron oxide nanoparticles, oxidative stress, apoptotic markers, histopathological alterations

## Abstract

The biochemical and histopathological changes induced by the exposure to iron oxide nanoparticles coated with phospholipid-based polymeric micelles (IONPs-PM) in CD-1 mice lungs were analyzed. After 2, 3, 7 and 14 days following the intravenous injection of IONPs-PM (5 and 15 mg Fe/kg bw), lactate dehydrogenase (LDH) activity, oxidative stress parameters and the expression of Bax, Bcl-2, caspase-3 and TNF-α were evaluated in lung tissue. An increase of catalase (CAT) and glutathione reductase (GR) activities on the second day followed by a decrease on the seventh day, as well as a decline of lactate dehydrogenase (LDH), superoxide dismutase (SOD) and glutathione peroxidase (GPx) activity on the third and seventh day were observed in treated groups *vs.* controls. However, all these enzymatic activities almost fully recovered on the 14th day. The reduced glutathione (GSH) and protein thiols levels decreased significantly in nanoparticles-treated groups and remained diminished during the entire experimental period; by contrast malondialdehyde (MDA) and protein carbonyls increased between the 3rd and 14th day of treatment *vs.* control. Relevant histopathological modifications were highlighted using Hematoxylin and Eosin (H&E) staining. In addition, major changes in the expression of apoptosis markers were observed in the first week, more pronounced for the higher dose. The injected IONPs-PM generated a dose-dependent decrease of the mouse lung capacity, which counteracted oxidative stress, thus creating circumstances for morphopathological lesions and oxidation processes.

## 1. Introduction

Over the last years, the nanomedicine field has made remarkable progress in developing tools for the diagnosis, prevention and treatment of diseases. Nanoparticles (NPs) possess optical, electronic and magnetic properties, which make them strong candidates for biomedical applications. Among the NPs, iron oxide ones are studied for their great potential in magnetic resonance imaging (MRI) [1], magnetic fluid hyperthermia (MFH), targeted drug delivery [2], biological labels [3], magnetic bio-separation and detection of biological molecules [4]. Although these NPs present good biocompatibility, as they are utilized by cells via iron metabolism pathways [5] previous studies have highlighted their cytotoxicity and possible *in vitro* [6,7,8,9,10] and *in vivo* [11,12,13] mechanisms involved in this phenomena. Nevertheless, due to high variations between different characteristics such as: size, shape, chemistry, surface and synthesis method, information regarding the IONPs toxicology is still lacking. In addition, depending on the biological environment, IONPs may undergo significant changes such as agglomeration or aggregation, disintegration or dissolution, as well as formation of surface coatings by proteins (biocorona) and other organic matter. Taking into account their use for human medical purposes, *in vivo* studies are of great importance for understanding their behavior in physiological systems. Previous studies proved that *in vitro* toxicology results were not well correlated with *in vivo* experiments [14,15] due to the involvement of body homeostasis. IONPs (ferric oxide, Fe_2_O_3_ or ferro-ferric oxide, Fe_3_O_4_) intravenously (IV) administrated in experimental animals, localize to the liver, spleen, kidneys, heart, brains, lungs, lymph nodes and bone marrow [16,17]. Accordingly, it was shown that more than 80% of the IV injected stabilized suspension of Fe_3_O_4_ NPs accumulated into liver, 10% into spleen while less than 2% were found in kidneys, lungs and heart following up to 14 days of exposure [18]. While it is thought that a large quantity of NPs introduced in the body would be cleared in urine in the first three days after administration [19], this might differ with particle size [12,20,21]. Taking into consideration the variations of NPs characteristics and their uncontrolled circulation within the human body, monitoring their effects on different tissues and organs is difficult. In the case of IV administrated NPs, the targeted organs are especially liver, spleen and kidneys, the lungs being ignored in most studies. Hanini *et al.* (2011) [19] detected inflammation in rat lungs, after IV injection of IONPs whereas Jain *et al.* (2008) [13] observed no significant changes in lipid hydroperoxide levels in the lung within three weeks following a similar treatment. Although a small quantity of IONPs reach the lungs, biochemical changes at the pulmonary level need to be investigated since the respiratory system is a receptor of the entire cardiac output and essential for life.

In this context, we were interested to study the modulation of oxidative stress parameters (antioxidant enzymes, reduced glutathione (GSH), malondialdehyde (MDA), protein oxidation products), the LDH activity, the histopathological alterations and the expression of some apoptotic related molecules, such as Bax, Bcl-2, caspase-3 and tumor necrosis factor α (TNF-α) induced by IV injection of IONPs-PM in CD-1 mice during 14 days of exposure.

## 2. Results

### 2.1. Characterization of Polymeric Micelles Loaded with Magnetic NPs

The oleic acid stabilized IONPs prepared by us present a hydrophobic surface. They were subsequently modified in order to become water-dispersable by encapsulation in the core of polymeric micelles (PM). Stable 1,2-Distearoyl-sn-glycero-3-phosphoethanolamine-*N*-[methoxy (poly(ethylene glycol))-2000] (ammonium salt) (DSPE-PEG) micelles encompassing magnetic material were obtained by mixing the IONPs with lipids during the micelle preparation. The size and surface potential of the micelles and IONPs are shown in Table 1.

**Table 1:** Size and zeta potential of the IONPs-PM.

Sample	Size (nm)	Zeta Potential (mV)
Pristine IONPs	12.5	–
DSPE-PEG micelles	14.9	−30.1
DSPE-PEG micelles loaded with IONPs	21.8	−28.7

IONP, iron oxide nanoparticles; DSPE-PEG, 1,2-Distearoyl-sn-glycero-3-phosphoethanolamine-*N*-[methoxy (poly(ethylene glycol))-2000].

The IONPs obtained were monodispersed (polydispersity index 0.086) with the average size of 12.5 nm, as observed from the dynamic light scattering (DLS) data. On the size distribution graph obtained for the iron oxide nanoparticles embedded in PEG-DSPE micelles (using DLS method) only one signal was present; no additional peak to be assigned to empty micelles was recorded.

**Figure 1:**
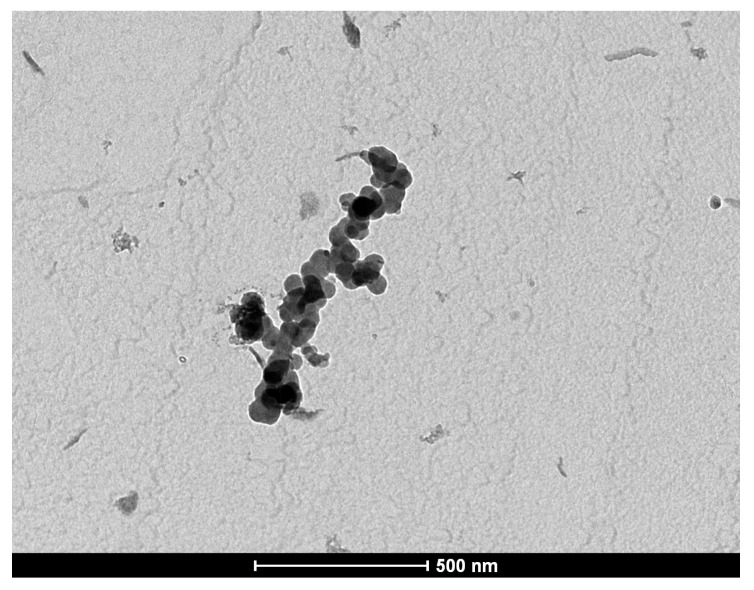
TEM image of polymeric micelles loaded with magnetic NPs. Scale bar = 500 nm.

In the Figure 1, a transmission electron microscopy (TEM) image is presented for the polymeric micelles loaded with IONPs.

The average size of the IONPs-PM was 21.5 nm, slightly higher than the size of unloaded (empty) micelles prepared from phospholipid polymeric derivative DSPE-PEG. The dimension and zeta potential of the DSPE-PEG micelles were similar to those previously reported in the literature [22,23].

The IONPs-PM produced an increase of the aggregate size. The surface potential of the unloaded and loaded DSPE-PEG micelles appeared similar.

The stability of IONPs-PM was evaluated by measuring the variation of the size (using the DLS method) during storage in normal conditions. The dispersion was stable, without any visible appearance of solid magnetic precipitate or variation of the micelle size for up to three months. The DSPE-PEG micelles loaded with IONPs were used in the experiment within two weeks of preparation.

### 2.2. Morphopathological Changes in Mice Lung Tissue

**Figure 2:**
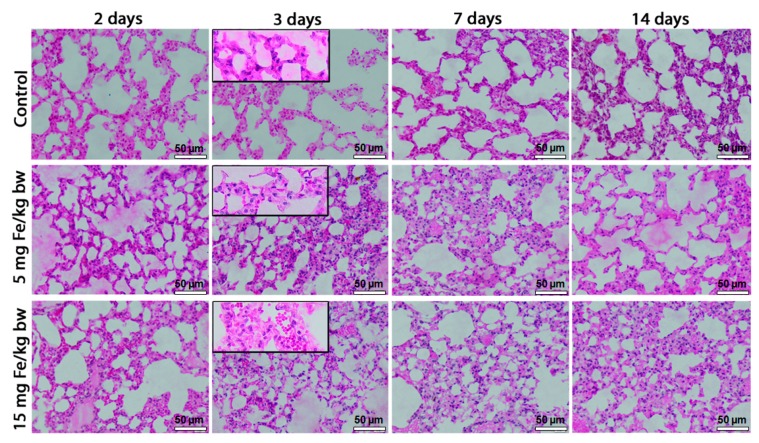
Histopathology of lung tissue after exposure to iron oxide nanoparticles coated with phospholipid-based polymeric micelles (IONPs-PM). Hematoxylin and Eosin (H&E) stain. Scale bar = 50 µm.

The lungs of IONPs-PM-exposed mice were characterized by a normal macroscopic appearance (smooth surface, shinning and marked out into numerous polyhedral areas, pinkish white colour) and were comparable with the control group. The histopathologic evaluation of the lung tissues by Hematoxylin and Eosin (H&E) staining showed a dose-dependent increase of pathological lesions, with maximum alterations on the third day of exposure for both doses, compared to control. We noticed increased extravasation of red blood cells into the lung parenchyma, inflammatory cells infiltration, thickening of the alveolar wall and the collapse of terminal bronchioles in mice exposed to the high dose of IONPs-PM (Figure 2).

### 2.3. Total LDH Activity

**Figure 3:**
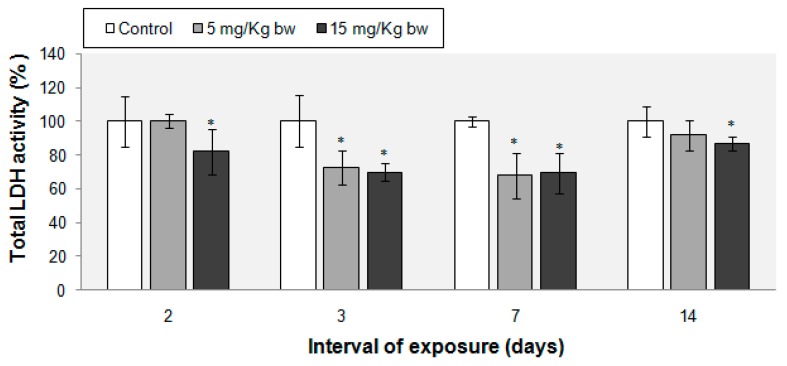
Changes of total LDH activity in mouse lung tissue exposed to IONPs-PM. Activity was calculated as means ± standard deviation (*n* = 7) and expressed as % from controls. * Means significantly different from controls (*p* < 0.05, α (significance level) = 0.05).

The LDH activity was measured from lung tissue homogenates for each time point. During the experiment, injected IONPs-PM induced a decrease of the total LDH activity compared with control groups. After two days of treatment, a change was noticed for the mice treated with 15 mg Fe/kg bw, the LDH activity being decreased by 18%. After three and seven days, we observed a significant reduction of LDH activity by 30% for both intervals of exposure and both doses of NPs. Conversely, an almost complete recovery of LDH activity was recorded on the 14th day of the experiment (Figure 3).

### 2.4. Activity of Oxidative Stress Related Enzymes

**Figure 4:**
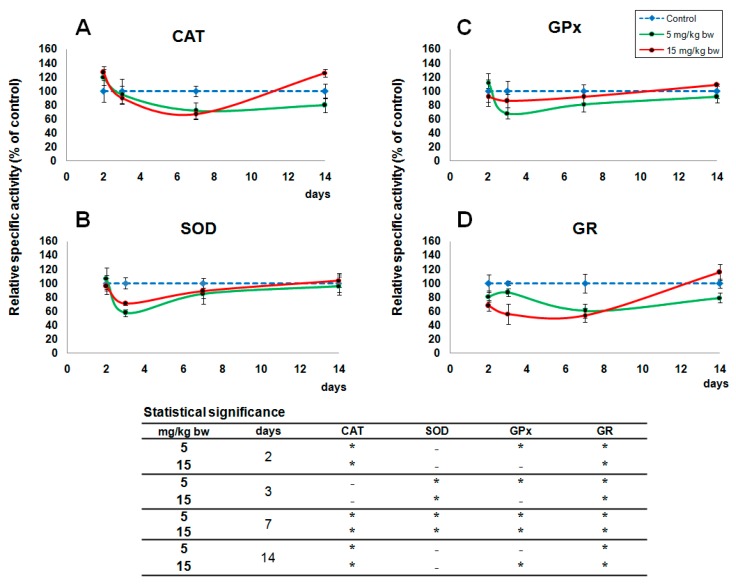
Effects of exposure to IONPs-PM on CAT (**A**); SOD (**B**); GPx (**C**); GR (**D**) in mice lung tissue. Activities are calculated as means ± standard deviation (*n* = 7) and expressed as % from controls. * Means significantly different from controls (*p* < 0.05, α (significance level) = 0.05).

The exposure of CD-1 mice to IV injected IONPs-PM induced significant changes in the specific activities of the antioxidant enzymes in the lungs (Figure 4). In the first two days after injection, the level of CAT activity increased for both treated groups (with 5 and 15 mg Fe/kg bw) by 20% and 26% respectively, compared to control. By contrast, CAT levels decreased until the 7th day of exposure, reaching 71% and 66% of control levels for both doses. After two weeks, the CAT activity remained 21% below the control for the group treated with the lower dose, whereas it continued to increase for the other experimental group, reaching a value of 125% of control.

The levels of GPx and SOD specific activities showed a similar pattern for both doses. SOD activity was not changed within two days of exposure, but starting with the third day, significant decreases were registered, by 43% and 30% below control for 5 and 15 mg Fe/kg bw doses, respectively. After the seventh day, these decreases were reduced to 16% and 12% of control, with these differences between the treated mice and control ones dissipating after two weeks. In the case of GPx activity, after the second day only the higher dose induced an increase of 10% whereas the lower one did not generated any change. But after the third day, a decrease by 32% and 9%, for the lower (5 mg Fe/kg bw) and respectively higher (15 mg Fe/kg bw) dose was observed and, starting with the seventh day of exposure GPx activity gradually increased and reached the control values after two weeks.

Our results also revealed a significant decrease in the activity of GR by 19% and 33%, for 5 and 15 mg Fe/kg bw doses starting with the first two days, which continued and reached the lowest value on the seventh day (40% and 46%). After two weeks, the level of GR activity in the group treated with 5 mg Fe/kg bw remained reduced (21% below control) but a significant increase was observed in the cohort treated with 15 mg Fe/kg bw (15% over control).

### 2.5. Variations in Reduced Glutathione (GSH) Content

**Figure 5:**
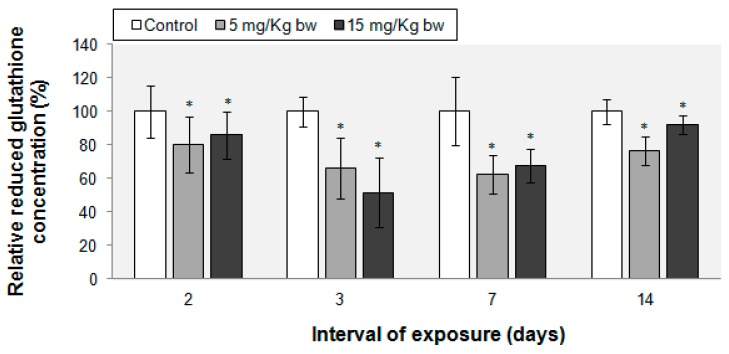
Reduced glutathione concentration in murine lung tissue after exposure to IONPs-PM. Data represent the relative mean ± standard deviation of seven animals per group. The values are expressed in percentages relative to control (100%). The difference between the treated group and the control group was significant when * *p* < 0.05, α (significance level) = 0.05.

We noted a significant decrease in the concentrations of the non-enzymatic antioxidant, GSH (Figure 5) starting with the second day following IV injection of IONPs (by 20% and 14% below control, respectively corresponding to 5 and 15 mg Fe/kg bw treated groups), which was maintained until the end of the experiment. After the third day the concentration of GSH was reduced by 34% and 49% respectively, with the trend continuing for the seven-day time point. However, after fourteen days, GSH levels started to increase, being diminished only by 24% and 8% for the groups treated with the low and high dose, respectively.

### 2.6. Lipid Peroxidation Evaluation

**Figure 6:**
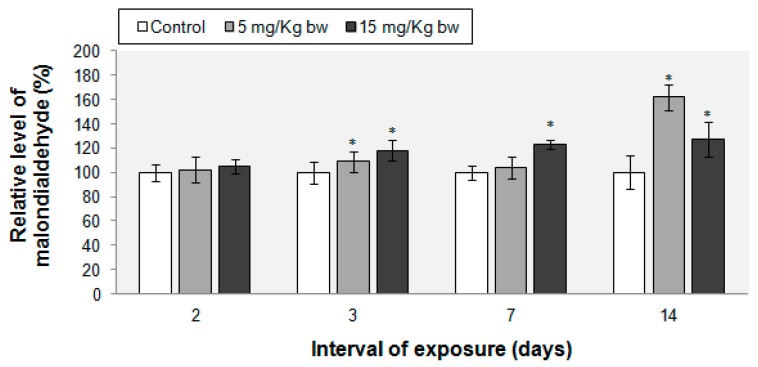
Malondialdehyde level in CD-1 mice lung tissue after exposure to IONPs-PM. Data represents the relative mean ± standard deviation of seven animals per group. The values are expressed in percentages relative to control (100%). The difference between the treated group and the control group was significant when * *p* < 0.05, α (significance level) = 0.05.

The peroxidation marker was unchanged after the first two days of exposure. After the 3rd, 7th and 14th day, the MDA level increased by 8% and 18%, 3% and 23%, respectively 61% and 27% in the groups treated with 5 and 15 mg Fe/kg bw (Figure 6).

### 2.7. Protein Oxidations Markers

**Figure 7:**
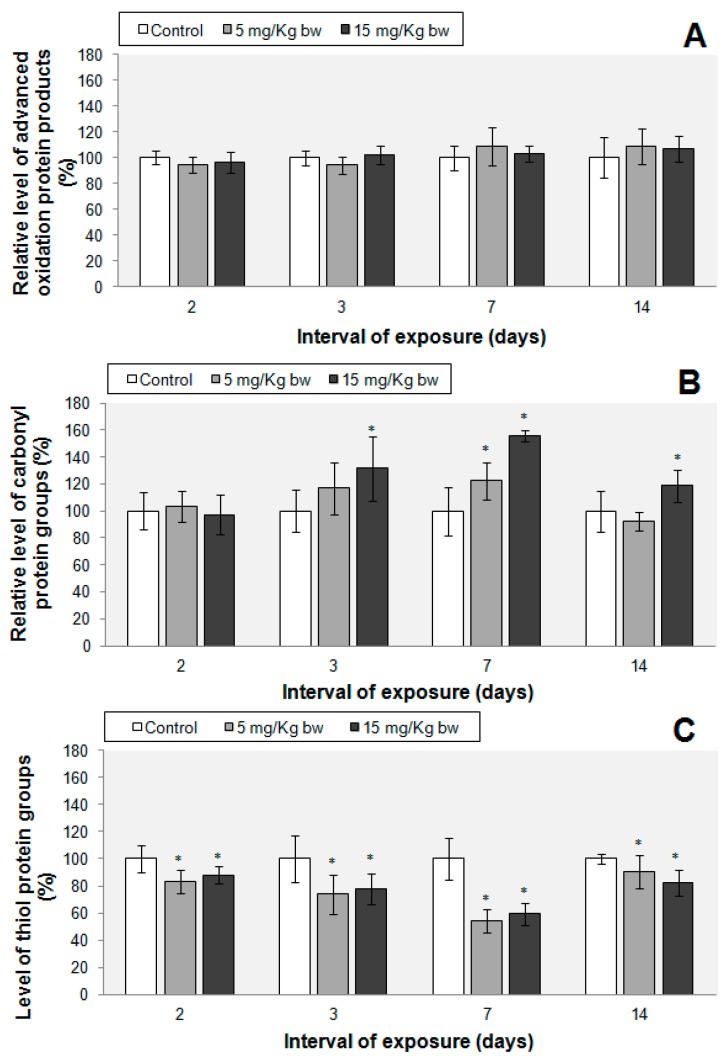
(**A**) Advanced oxidation protein products; (**B**) Protein carbonyl; (**C**) Protein thiol groups levels in murine lung tissue after exposure to phospholipid micelles IONPs. Data represent the relative mean ± standard deviation of seven animals per group. The values are expressed in percentages relative to control (100%). The difference between the treated group and the control group was significant when the * *p* < 0.05, α (significance level) = 0.05.

The concentration of AOPP did not change significantly up to the 7th day of exposure (Figure 7). A significant change in AOPP content by 28% was noticed only after the second week, in the group treated with 15 mg Fe/kg bw. High levels of protein carbonyl groups were observed only in the 3rd, 7th and 14th days of exposure, in the 15 mg Fe/kg bw treated group, by 31%, 55% and 18% respectively, above control. In the 5 mg Fe/kg bw treated group, the concentration of protein carbonyl groups was significantly increased only on the 7th day of exposure (by 22% above control). By contrast, thiol levels were lower in the IONPs-PM-treated groups throughout the whole experiment reaching the minimum concentration on the seventh day (47% and 41% below control, corresponding to the 5 and 15 mg Fe/kg bw treated groups).

### 2.8. Evaluation of Apoptotic Markers

**Figure 8:**
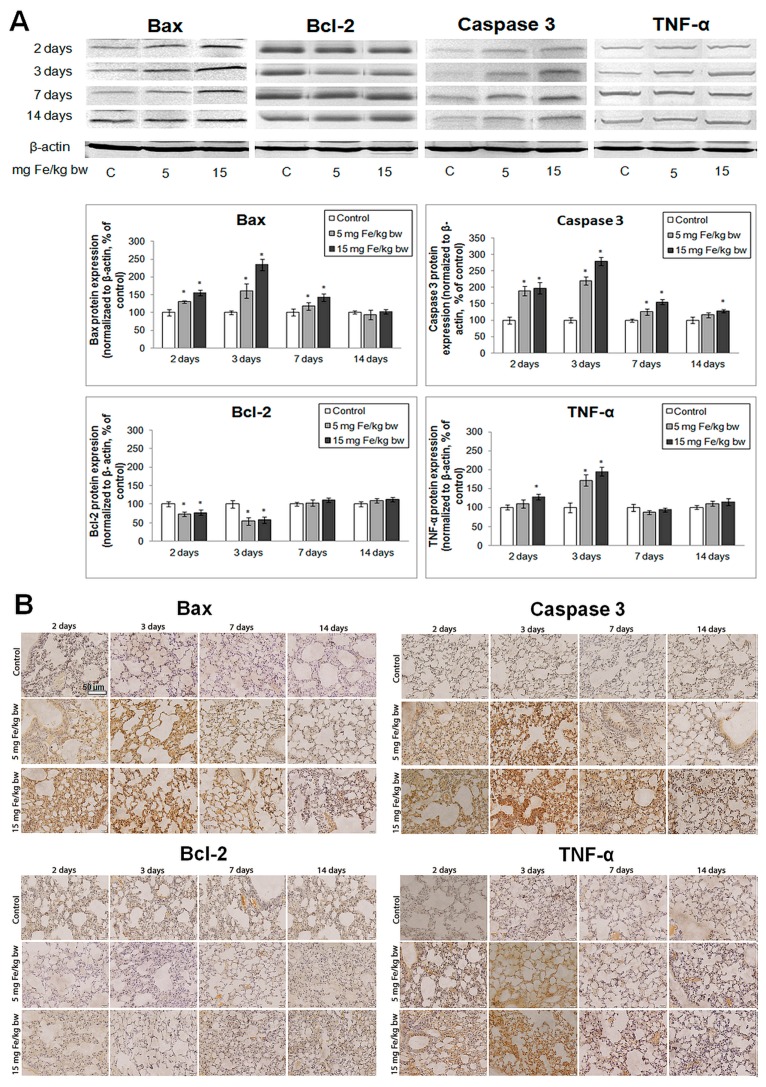
Expression of Bax, Bcl-2, Caspase-3 and TNF-α in the mouse lung tissue after IONPs-PM administration. (**A**) Western blot analysis (blot images and bands quantification) and (**B**) immunohistochemistry (IHC). Scale bar is 50 µm and is the same for all IHC images. Notes: For IHC and Western blot a representative image was chosen. The values obtained from bands quantification were calculated as relative mean ± standard deviation of four animals per group. The difference between the treated group and the control group was significant when * *p* < 0.05, α (significance level) = 0.05. Data were normalized to β-actin and represented as % of control.

Protein levels of Bax, Bcl-2, active caspase-3 and TNF-α were examined by immunohistochemistry (IHC) and Western blot analyses. Blotting analyses (Figure 8A), revealed that IONPs-PM induced a significant increase in pro-apoptotic proteins expression (Bax and active caspase-3) during the first three days of exposure and a simultaneous down-regulation of Bcl-2 expression. Furthermore, in the first three days, an increase in TNF-α protein expression was observed. A recovery was noticed for all studied proteins to values close to the control starting with the 7th day through to the end of the experiment. These results were sustained by IHC images (Figure 8B) that showed a similar pattern.

## 3. Discussion

Toxicological mechanisms induced by IONPs are widely studied due to their importance for the medical field. Currently, it is well known that *in vitro*, intracellular dissolution of uncoated IONPs could affect cell homeostasis and cause toxicity via oxidative stress, inflammation and genotoxicity [24]. However, many of the toxic responses seen *in vitro* are not reproduced *in vivo* due to the homeostasis maintained by the liver and kidneys which may regulate any changes that occur in the blood plasma parameters (pH, ionic strength and chemical composition) [25,26]. The determination of the *in vivo* mechanisms of toxicity is more challenging due to the strong connection between organ systems and the influence of the biological environment; however, these analyses have a major significance for the design of biocompatible NPs and for understanding the non-specific interactions with different tissues, as well as their distribution and clearance in the body [27].

When covered with an adequate coating (e.g., silicon, dextran, citrate, polyethylene glycol), IONPs become more biocompatible [28] and give different intracellular responses. In the current study, phospholipid polymeric DSPE-PEG (1,2-Distearoyl-sn-glycero-3-phosphoethanolamine-*N*-methoxy (poly(ethylene glycol))-2000) micelles have been chosen for the encapsulation of magnetic IONPs due to the fact that PM have a series of beneficial properties such as *in vitro* and *in vivo* high stability, good biocompatibility, and the ability to uptake a variety of poorly soluble chemicals. Also, this type of encapsulation determined increased circulation time in the bloodstream, good dispersion in biological fluids, limited protein adsorption, prevention of phagocytosis and improved biocompatibility [29,30]. Recently, it was proved that PEG coated NPs, can be employed for MRI scanning within 12 days after administration, which is important for clinical diagnosis [17].

*In vivo*, IONPs can be degraded into iron ions which are metabolized in the liver by Kupffer cells and in the spleen by macrophages from the red pulp, with a part of them accumulating into tissues and leading to cellular responses. Once present in the cell, iron can be sequestered within ferritin, which limits its capacity to generate free radicals [31]. However, when ferritin is overloaded, free iron ions could be released into the cell where they can react with hydrogen peroxide produced by the mitochondria, microsomes, peroxisomes and cytosolic enzymes respectively, and generate highly reactive hydroxyl radicals and ferric ions (Fe^3+^) via the Fenton reaction [32]. For MRI studies a dose of 100 µmoles Fe/kg bw is often used [33], which corresponds to about 5 mg Fe/kg bw. Taking this into account, we have chosen two doses in the same range. As a result, our data could provide useful information for clinical practice.

The cells are protected against these negative effects by a variety of defense systems including enzymatic antioxidant mechanisms (CAT, SOD, GPx, GR) and non-enzymatic antioxidants (GSH, vitamins, albumin, uric acid, bilirubin, *etc.*). There are considerable differences between the activities of antioxidant enzymes in different tissues due to metabolic specialization, the presence of various environmental factors, the degree of oxygenation or the exposure to specific metabolites [34].

Due to their anatomy, localization and function, lungs are permanently exposed to oxidation given their high oxygen usage. This has lead to the evolution of an antioxidant defense system to protect them against substantial damage [35]. In inflammatory situations involving this organ, great amounts of hydrogen peroxide and superoxide are released by activated macrophages and neutrophils via the phagocytic isoforms of nicotinamide adenine dinucleotide phosphate (NADPH) oxidase. Additionally, the non-phagocytic cells produce ROS at the level of mitochondria and microsomes [35,36].

Iron released from IONPs may be another source of ROS generation. In oxidative stress conditions, respiratory epithelial cells and alveolar macrophages should be unable to reestablish iron equilibrium and the iron ions could be metabolized by neutrophils and stored within ferritin. Furthermore, neutrophils may generate reductants (e.g., ascorbate and superoxide) that can reduce Fe^3+^ to Fe^2+^ and exacerbate the level of oxidative stress [31]. Recently, it was demonstratedthat IONPs treatment induces a loss of mitochondrial membrane potential [37] that can be correlated with increased ROS production [38].

*In vitro* ROS production induced by bare IONPs was presented in several toxicity studies on pulmonary cells [7,8,39]. In a previous work, we observed that after two days of exposure, 12.5 µg/mL of bare IONPs have the capacity to increase ROS production in MRC-5 cells by 52% compared to control. For longer periods of exposure, ROS levels remained up-regulated, but to a lesser extent. In accordance with our study, Ahmed *et al.* (2013) [40] exposed lung epithelial A549 cells to 25–100 µg/mL IONPs for 24 h and noticed a dose-dependent increase in ROS production by 55% to 130% respectively compared to control. Compared to these findings, IONPs covered with layers of hydrophilic and biocompatible polymers showed slight but essential changes regarding their capacity to induce cytotoxic effects. Prevoiusly, it was assumed that the coating helped to minimize the reactivity of the IONPs resulting in less ROS generation. We have demonstrated that a similar dose of iron oxide nanoparticles coated with phospholipid-based polymeric micelles induced a lower level of ROS *in vitro* than bare IONPs (by 24% after 72 h of exposure) (unpublished data). The mechanism by which nanoparticles generate ROS might differ depending on coating properties. Research conducted by Yu *et al.* (2012) [41] highlighted that polymer coatings can decrease ROS-induced nanoparticle toxicity depending on nanoparticle size. By contrast, Chen *et al.*, 2012 [42] indicated that polymer-coated IONPs (2 mg/mL) had a higher toxicity than uncoated IONPs, which led to an increase of 0.2-fold in ROS generation.

In our case, a significant increase of CAT activity was noticed two days post IONPs-PM IV injection in mice exposed to both doses, with the GPx activity being raised to a lesser extent. Beyond the reaction catalyzed by SOD, hydrogen peroxide can be formed at the level of complexes I and III of the respiratory chain [43] and by soluble enzymes of the matrix [44]. For the same time interval, a significant decrease in GR activity was noticed for both doses.

It would appear that an acute state of oxidative stress was established on the third and seventh days when a general decrease of antioxidant enzymes (CAT, SOD, GPx, GR) occurred.

When oxidative stress persists for several days, the mitochondrial engine can suffer damages (*i.e.*, the dysregulation of the electron transport can lead to increased mitochondrial generation of H_2_O_2_) leading to more oxidative stress and the decrease of ATP production. The decrease in enzymatic activities may occur via direct oxidative damage of the molecules or via alteration of the antioxidant enzyme gene expression. In addition, the variation of GSH concentration followed the same pattern as that of GR activity, reaching the minimum values on the third and seventh days. Also, no major differences were observed between doses. It is possible that in this time interval, GR did not catalyze the formation of GSH from G–S–S–G and NADPH and as a result, lipid peroxidation occurred. These data are in agreement with those obtained by Popescu *et al.* (2015) [45] for the mice spleen.

After the 14th day, the enzymatic activities of SOD, CAT and GPx were restored and the cells probably acquired a better protection system. In contrast with our results, Zhu *et al.* (2008) [46] reported an increase in the antioxidant protection after just seven days of exposure to Fe_2_O_3_. Many studies showed that ROS production plays an important role in the regulation of some molecular pathways that lead to the induction of lung diseases. Thus, oxidative stress has been correlated with various lung disorders such as: asthma, chronic obstructive pulmonary disease (COPD), acute lung injury, pulmonary fibrosis and lung cancer [47,48,49].

Lipid peroxidation is a process in which ROS attack lipids, especially polyunsaturated fatty acids, and primary (lipid hydroperoxides) and secondary products (malondialdehyde, propanal, hexanal, and 4-hydroxynonenal) are formed. In the first two days after IONPs-PM injection, no lipid peroxidation was observed probably due to the increased CAT and GPx activities. Nevertheless, beginning with the third day, a raise of the MDA level in lung tissue exposed to both doses occurred as a result of the decrease of the lung’s antioxidant activity. Similar results were found in a four week study conducted by Gaharwar and Paulraj (2015) [50], where lipid peroxidation levels in peripheral blood cells increased in a dose and time dependent manner for 7.5, 15 and 30 mg/kg of IONPs, starting with the second week of treatment.

Protein oxidation as a result of oxidative stress may occur either directly or as a consequence of lipid peroxidation [51]. In our study, the most significant changes were noticed in the third and seventh day. High levels of carbonyl protein groups were present until the 14th day in accordance with the induction of lipid peroxidation. Protein carbonyls can be formed in many ways: by oxidative cleavage of the protein backbone, direct oxidation of amino acids (lysine, histidine, arginine, proline, threonine and glutamic acid) or the binding of aldehydes produced by lipid peroxidation. Also, carbonyl groups may be added to proteins by reactions with reactive carbonyl derivatives generated as a consequence of the reducing sugars reactions or their oxidation products with lysine residues of proteins, glycation or glycoxidation reactions [52].

The level of thiol protein groups registered a decline beginning with the second day, which was maintained until the end of the experiment, suggesting that they were involved in ROS counteraction, with no significant changes being observed in AOPP levels.

The inactivation of antioxidant enzymes and an enhanced ROS production may result in cell death [53].

Our data support the hypothesis that oxidative stress induced by IONPs-PM can result in the morphological alteration of the pulmonary tissue, especially when mice were treated with higher concentration of NPs. This change consisted in the increased extravasation of red blood cells, inflammatory cells infiltration and thickening of the alveolar wall, with maximum changes at three days of exposure. LDH activity is another indicator of pulmonary injury, because of its release by cytolysis [54]. We have noticed a 30% decrease of the LDH activity in mice lung tissues on the third and seventh day following IONPs-PM injection, probably as a consequence of its release into the bloodstream.

Our results are also supported by the previous findings suggesting that the cytotoxicity of NPs mediated through cellular stress induced morphological alterations in A-549 cells exposed to IONPs [55].

In this context, we have found an increase in TNF-α and pro-apoptotic Bax expressions, activation of caspase-3 as well as Bcl-2 down-regulation. Additionally, we observed that the changes of these proteins expression were more pronounced for the higher dose. Apoptosis plays an important role in normal lung homeostasis and is involved in the pathogenesis of a variety of lung diseases and may be induced by many signaling pathways [56,57]. Previous studies have reported that apoptosis (both intrinsic and extrinsic pathways), a major mechanism of cell death, is induced by nanoparticles in oxidative stress conditions [58,59,60]. It is well known that TNF-α is a potent inducer of apoptosis but at the same time it can regulate survival and proliferation mechanisms via the activation of NF-κB-dependent genes. Induction of apoptosis by TNF-α is primarily caused by the activation of type I receptors (including Fas/CD95) and caspases [61]. In our study, IONPs-PM induced the up-regulation of TNF-α in lung tissue on the second and third days post IV injection, suggesting an activation of the apoptosis process. At the same time, Bax expression increased and caspase 3 was activated. Conversely, Bcl-2 expression was significantly inhibited in the first three days of exposure. However, after 14 days, the protein expression levels of all analyzed molecules were close to the control values, suggesting that the apoptosis process was inhibited. GSH depletion is a known contributing factor to the disruption of the mitochondrial phospholipids membrane and its depolarization [62]. Permeabilization of the outer mitochondrial membrane by Bax, releases cytochrome c into the cytosol and leads to caspase-3 activation. Taking into account our results, we suggest that IONPs-PM initially triggered apoptotic death in a ROS dependent manner in the mouse lung tissue for the first three days of exposure, with this tendency being efficiently counteracted by the antioxidant system at later time points.

Animal studies are suited for observing the overall effects of an experiment on living subjects. In our case, we intended to compare the short-term benefit due to IONPs accumulation in the lung, which offers the possibility to use MRI, with midterm harm. Our data are useful, but as it was previously observed they could be different compared to those obtained in the human trials [63]. Although mice and humans are at least 95% identical at the genomic level, their phenotypes are very different [64]. However, animal models are still an important source of *in vivo* information, which represent a bridge between *in vitro* studies and human patients.

## 4. Experimental Section

### 4.1. Reagents

Modified phospholipid 1,2-Distearoyl-sn-glycero-3-phosphoethanolamine-*N*-[methoxy (poly(ethylene glycol))-2000] (ammonium salt) (DSPE-PEG) was supplied by Avanti Polar Lipids Inc. (Alabaster, AL, USA) whereas iron(III) acetylacetonate (Fe(acac)_3_), 1,2-hexadecanediol (90%), oleic acid (90%), oleylamine (70%), phenyl ether (99%) were purchased from Sigma-Aldrich (St. Louis, MO, USA) and used without further purification. Specific primary antibodies against Bax (mouse monoclonal), Bcl-2 (mouse monoclonal), TNF-α (goat polyclonal,) active caspase-3 (rabbit polyclonal) and β-actin (mouse monoclonal) proteins were purchased from Santa Cruz Biotechnology, Inc. (Heidelberg, Germany). All other chemicals were of analytical grade and were purchased from standard commercial suppliers.

### 4.2. Synthesis of Magnetic IONPs

IONPs were prepared using a simple solvothermal method with minor modifications [65]. Briefly, a mixture of Fe(acac)_3_ (2 mmol), hexadecanediol (6 mmol), oleic acid (6 mmol) and oleylamine (6 mmol) in phenyl ether (30 mL) was placed into a 100 mL three-necked flask, under vigorous stirring, in a current of nitrogen. The solution was heated to 280 °C, and the reaction temperature and stirring were maintained for 3 h. The solution color changed to black, indicating the formation of magnetite nanoparticles. Finally, the solution was cooled to room temperature by removing the heat source. IONPs were precipitated by the addition of excess ethanol and were subsequently subjected to centrifugation (4500 rpm, 10 min). The solid precipitate was washed three times with ethanol to remove the reactants. The precipitated nanoparticles were re-dispersed into chloroform and were ready to be used for the encapsulation in polymeric micelles.

### 4.3. Preparation of Polymeric Micelles Loaded with IONPs

The encapsulation of IONPs in the polymeric micelles was performed using the dry film hydration method. The phospholipid polymeric derivative DSPE-PEG together with a certain amount of hydrophobically modified IONPs was dissolved in a minimal volume of chloroform into a round bottom flask. The solvent was then evaporated under reduced pressure to produce a homogeneous film onto the walls of the vial, using a rotaevaporator. The resulting lipid film containing IONPs was hydrated by adding 0.9% saline solution to obtain the final concentrations of 20 mg/mL polymer and 1000 μg/mL IONPs. The so prepared micellar dispersion was sonicated for 5 min at room temperature and finally filtered through a Millex filter (0.22-μm-diameter, Merck KGaA, Darmstadt, Germany).

### 4.4. Characterization of Polymeric Micelles Loaded with IONPs

The size distribution of the IONPs covered by polymeric micelles was measured using the dynamic light scattering DLS method. The determinations were performed on a Zetasizer Nano-ZS90 Malvern Instruments (Malvern Instruments Ltd., Malvern, UK). The particle zeta potential was calculated by Laser Doppler Velocimetry (LDV). The IONPs dimensions were confirmed by TEM. For the TEM measurements the samples were prepared from chloroform dispersion spread onto a carbon holey grid, dried at room temperature and analyzed with a TECNAI-20 electron microscope FEI instrument (FEI Company, Eindhoven, The Netherlands). To verify the stability of IONPs-loaded polymeric micelles the samples were stored at 25 °C in normal light for three months.

### 4.5. Experimental Animals and Treatment

Male CD1 mice (12–14 weeks old) weighing 20–30 g were bred and maintained in normal watering and feeding conditions in a Laboratory Animal Husbandry Facility, equipped with IVC (individually ventilated cages), controlled atmosphere-temperature, humidity and lighting (Vasile Goldis Western University, Arad, Romania). All experimental procedures were in accordance with the bioethical rules approved by the Institutional Ethical Committee. Mice were selected by randomization and divided into 3 groups of twenty eight individuals (4 subgroups/group with *n* = 7) as follows: (1) the control grou: mice injected with 0.9% NaCl in the tail vein; (2) the treated group 1: mice injected with suspensions of IONPs corresponding to 5 mg Fe/kg bw in the tail vein; (3) the treated group 2: mice injected with suspensions of IONPs corresponding to 15 mg Fe/kg bw in the tail vein. For each group, 7 mice were sacrificed after 2, 3, 7 and 14 days. During the experiment no significant change of mice weight was registered. Lung samples were collected and used for histopathology, immunohistochemistry and biochemical analyses.

### 4.6. Histopathology

Small pieces of lung tissue were fixed in Bouin’s solution (24 h), dehydrated in ethanol, cleared in toluene and embedded in paraffin wax. Five µm-thick sections were stained with H&E and analyzed by light microscopy using the Olympus System Microscope Model BX43 (Olympus Europa SE & CO, Hamburg, Germany) equipped with a digital camera (Olympus XC30, Olympus Europa SE & CO, Hamburg, Germany).

### 4.7. Tissue Extract Preparation and Protein Determination

For the preparation of total protein extract, 0.1 g of tissue was homogenized in 1 mL of 0.1 M Tris/EDTA buffer, pH 7.4 by sonication. After centrifugation at 10,000 rpm, 4 °C for 30 min, the supernatant was aliquoted and stored at −80 °C for further investigations. Total protein content was determined according to Lowry’s method (1951) [66] using bovine serum albumin as standard.

### 4.8. Measurement of Total LDH Activity

Total LDH activity was estimated following the spectrophotometric method of Borgman *et al.* [67]. Briefly, accordingly diluted tissue extracts and the reaction medium containing 0.2 M Tris, pH 7.3, 6.6 mM NADH and 30 mM sodium pyruvate were mixed and the reaction velocity was determined by the decrease in absorbance of NADH at 340 nm for 5 min. One unit of LDH activity causes the oxidation of one µmole of NADH per minute at 25 °C. The LDH activity was calculated as relative specific activity (U/mg of protein) and expressed as % of controls.

### 4.9. Enzymatic Activities

Catalase (CAT) activity was evaluated by monitoring the reduction of H_2_O_2_ at 240 nm [68]. Superoxide dismutase (SOD) activity was determined by measuring the decrease in NADPH after the oxidation of superoxide at 340 nm [69]. Glutathione peroxidase (GPx) activity was assayed by measuring the conversion of NADPH to NADP^+^ at 340 nm, following the reduction of GSSG to GSH mediated by glutathione reductase [70]. Glutathione reductase (GR) activity was determined by recording the decrease in NADPH concentration at 340 nm [71]. All the enzymatic activities were calculated as relative specific activities (U/mg of protein) and expressed as % of controls.

### 4.10. Reduced Glutathione Quantification

The GSH content was evaluated using the Glutathione Assay Kit from Sigma-Aldrich according to the manufacturer’s instructions. For GSH evaluation, protein extracts were first treated with 5-sulfosalicylic acid. The supernatants were added in a reaction mix containing enzyme solution, DTNB and assay buffer and incubated for 5 min at room temperature. After adding the NADPH solution, TNB formed was measured spectrophotometrically at 405 nm. The results were expressed in nmoles GSH/mg of protein and presented as % of controls.

### 4.11. Lipid Peroxidation Measurement

The level of malondialdehyde (MDA), as a marker of lipid peroxidation was assessed using the method described by Dinischiotu *et al.* (2013) [72]. Briefly, 200 µL of lung tissue extract were mixed with 700 µL 0.1 N HCl and incubated for 20 min at room temperature. A volume of 900 µL of 0.025 M thiobarbituric acid (TBA) was then added and the total volume was maintained for 65 min at 37 °C. The MDA level in tissue samples was measured at 520/549 nm (excitation/emission) using 1,1,3,3-tetramethoxypropane as standard. The results were calculated as nmoles of MDA/mg of protein and expressed as % of controls.

### 4.12. Proteins Oxidation Assays

- Advanced oxidation protein products (AOPP) levels in the mouse lung tissue were evaluated according to the method of Witko *et al.* (1992) [73]. Briefly, a volume of 200 µL of protein extract was mixed with 10 µL of 1.16 M KI in a 96-well plate and allowed to stand for 5 min at room temperature. Subsequently, 20 µL of glacial acetic acid were added and, after 10 min, the optical density was read at 340 nm. Chloramine T was used as a standard. The results were calculated as µmoles of AOPP/mg of protein and expressed as % of controls.
- Protein carbonyl groups (CO) levels were measured according to the Fields and Dixon’s method (1971) [74], which is based on the reaction of 2,4-dinitrophenylhydrazine (DNPH) with protein carbonyls resulting in hydrazones. A volume of 500 µL protein extract was incubated with 500 µL of 10 mM DNPH (in 2 M HCl), for 1 h, at room temperature. The proteins were then precipitated with 500 µL ice-cold 20% TCA and centrifuged at 13,000 rpm for 3 min. The pellets were washed three times with 500 µL ethanol:ethyl acetate mixture and dissolved in 600 µL 1 M NaOH. The samples absorbance was read at 370 nm and the concentration expressed in nmoles CO/mg protein was calculated using a molar extinction coefficient of 22.000 M^−1^·cm^−1^. Finally the concentrations were expressed as % of controls.
- Protein sulfhydryl groups (-SH) were determined according to the method of Riener *et al.* (2002) [75]. Briefly, the protein extract (100 µL) was deproteinized with an equal volume of 20% TCA and centrifuged for 10 min at 10,000 rpm, at 4 °C. The pellet was dissolved in 20 µL of 1 M NaOH. Before reading the optical density at 324 nm, the soluble pellets were incubated for 5 min with 730 µL of 0.4 M Tris-HCl buffer, pH 9 and 30 µL of 4 mM 4,4′-dithiodipyridine (DTDP). The concentration of protein sulfhydryl groups (nmoles/mg of protein) was quantified using a *N*-acetyl-cysteine standard curve and expressed as % of controls.

### 4.13. Immunological Techniques

#### 4.13.1. Immunohistochemistry

After deparaffinization in toluene, paraffin-embedded mouse lung sections were re-hydrated in graded series of ethanol (100%, 96%, 70%), washed under running water and clarified with distilled water. The immunohistochemistry evaluation was accomplished using the Max Polymer Detection System (Novo Link Kit, Novocastra^®^, Milton Keynes, UK). The slides were incubated with the reagents supplied by the kit following the procedures and incubation times suggested in the manufacturer’s specifications. Thus, endogenous peroxidase activity was neutralized using the Peroxidase Block and the unspecific binding was reduced by adding the Novocastra™ Protein Block. Sections were incubated overnight in a sealed humidity chamber with primary antibodies for Bax, Bcl-2, cleaved caspase-3 and TNF-α (1:100 dilution) and then specific secondary antibodies (Post Primary) were applied. Sections were further incubated with the chromogen substrate, 3,3′-diaminobenzidine (DAB) which produces a visible brown precipitate in a reaction with the peroxidase at the antigen site and counterstained with hematoxylin. After dehydratation, tissue sections were mounted with BioMount mounting medium and analyzed by light microscopy (20× magnification) using the Olympus System Microscope Model BX43 equipped with a digital camera. Five stained slides of each group were visualized and five views from random fields were evaluated for each slide.

#### 4.13.2. Immunoblot Analysis

Protein extracts obtained from mouse lung tissue (40 µg) were denatured at 95 °C for 5 min in sample buffer and loaded on 10% and 15% denaturing polyacrylamide gels using the Bio-Rad Mini-PROTEAN system. Separation of proteins was performed at 70–90 V in 1× running buffer (0.05 M TRIS, 0.05 M Glycine and 0.1% SDS). Then, proteins were transferred for 1.5 h at 350 mA (Tris-glycine buffer: 25 mM TRIS, 192 mM glycine, pH = 8.3, with 20% methanol) to a polyvinylidenedifluoride (PVDF) membrane using a wet electroblotting system (Bio-Rad Laboratories, Hercules, CA, USA). The membranes were developed using the Western Breeze Chromogenic Immunodetection Kit (Life Technologies, Darmstadt, Germany) following the manufacturer’s instructions. After 30 min in blocking solution, membranes were incubated for 1 h at room temperature with specific monoclonal primary antibodies against Bax, Bcl-2, TNF-α and active caspase-3. β-Actin was used as reference protein. Appropriate secondary antibodies conjugated with alkaline phosphatase were added for 30 min before staining with BCIP/NBT substrate. Protein bands were visualized with the Bio-Rad ChemiDoc Imaging System (Bio-Rad, Hercules, CA, USA) and protein expression was quantified with the Bio-Rad Image Lab software (version 5.2, Bio-Rad, Hercules, CA, USA). Data were normalized to β-actin and protein expressions were represented as percentage from controls.

### 4.14. Data Analysis

All experiments were carried out in triplicate and seven animals were used per group. Data were compared using student’s *t*-test (two-tailed, unpaired) and validated by confidence intervals (α = 0.05, level of confidence is 95%) for control and treated cells samples using the Quattro Pro X7 software (Corel, Ottawa, ON, Canada). Values were expressed as mean ± standard deviation (SD). The results were considered statistically significant when * *p* < 0.05.

## 5. Conclusions

The extended time that NPs reside in the body is of great concern to human health. Our results underline the importance and necessity of toxicological experiments before prospective clinical use. In this study we have shown histopathological and biochemical modifications in lung tissue for a period of 14 days after IONPs-PM IV injection. These changes were triggered by oxidative stress and apoptosis in a time and dose dependent manner. Although the biochemical and structural alterations diminished after three days of exposure, the persistence of biomolecules oxidation was noticed up to 14 days. Our data suggest that while IONPs-PM could be used for diagnostic (e.g., MRI) and therapeutic applications, further investigations are needed prior to condoning wide spread use.

## References

[1] Jin R., Lin B., Li D., Ai H. (2014). Superparamagnetic iron oxide nanoparticles for MR imaging and therapy: Design considerations and clinical applications. Curr. Opin. Pharmacol..

[2] Lin M.M., Kim D.K., El Haj A.J., Dobson J. (2008). Development of superparamagnetic iron oxide nanoparticles (SPIONS) for translation to clinical applications. IEEE Trans. Nanobiosci..

[3] Soenen S.J., Himmerlreich U., Nuytten N., Cuyper M.D. (2011). Cytotoxic effects of iron oxide nanoparticles and implications for safety in cell labelling. Biomaterials.

[4] Huang S.H., Juang R.S. (2011). Biochemical and biomedical applications of multifunctional magnetic nanoparticles: A review. J. Nanopart. Res..

[5] Bulte J.W., Kraitchman D.L. (2004). Iron oxide MR contrast agents for molecular and cellular imaging. NMR Biomed..

[6] Huang Y.W., Wu C.H., Aronstam R.S. (2010). Toxicity of transition metal oxide nanoparticles: Recent insights from *in vitro* studies. Materials.

[7] Mahmoudi M., Simchi A., Imani M., Shokrgozar M.A., Milani A.S., Häfeli U.O., Stroeve P. (2010). A new approach for the *in vitro* identification of the cytotoxicity of superparamagnetic iron oxide nanoparticles. Colloids Surf. B Biointerfaces.

[8] Radu M., Dinu D., Sima C., Burlacu R., Hermenean A., Ardelean A., Dinischiotu A. (2015). Magnetite nanoparticles induced adaptative mechanisms counteract cell death in human pulmonary fibroblasts. Toxicol. In Vitro.

[9] Naqvi S., Samim M., Abdin M.Z., Ahmed F.J., Maitra A.N., Prashant C.K., Dinda A.K. (2010). Concentration-dependent toxicity of iron oxide nanoparticles mediated by increased oxidative stress. Int. J. Nanomed..

[10] Karlsson H.L., Holgersson A., Moller L. (2008). Mechanisms related to the genotoxicity of particles in the subway and from other sources. Chem. Res. Toxicol..

[11] Takeda K., Suzuki K.I., Ishihara A., Kubo-Irie M., Fujimoto R., Tabata M., Oshio S., Nihei Y., Ihara T., Sugamata M. (2009). Nanoparticles transferred from pregnant mice to their offspring can damage the genital and cranial nerve systems. J. Health Sci..

[12] Gu L., Fang R.H., Sailor M.J., Park J.H. (2012). *In vivo* clearance and toxicity of monodisperse iron oxide nanocrystals. ACS Nano.

[13] Jain T.K., Reddy M.K., Morales M.A., Leslie-Pelecky D.L., Labhasetwar V. (2008). Biodistribution, clearance, and biocompatibility of iron oxide magnetic nanoparticles in rats. Mol. Pharm..

[14] Sayes C.M., Reed K.L., Warheit D.B. (2007). Assessing toxicity of fine and nanoparticles: Comparing *in vitro* measurements to *in vivo* pulmonary toxicity profiles. Toxicol. Sci..

[15] Szalay B. (2012). Iron Oxide Nanoparticles and Their Toxicological Effects: *In vitro* and *in vivo* Studies. Ph.D. Thesis.

[16] Zhuang J., Fan K., Gao L., Lu D., Feng J., Yang D., Gu N., Zhang Y., Liang M., Yan X. (2012). *Ex vivo* detection of iron oxide magnetic nanoparticles in mice using their intrinsic peroxidase-mimicking activity. Mol. Pharm..

[17] Ruiz A., Hernández Y., Cabal C., González E., Veintemillas-Verdaguer S., Martínez E., Morales M.P. (2013). Biodistribution and pharmacokinetics of uniformmagnetite nanoparticles chemically modified with polyethylene glycol. Nanoscale.

[18] Ma H.L., Xu Y.F., Qi X.R., Maitani Y., Nagai T. (2008). Superparamagnetic iron oxide nanoparticles stabilized by alginate: Pharmacokinetics, tissue distribution, and applications in detecting liver cancers. Int. J. Pharm..

[19] Hanini A., Schmitt A., Kacem K., Chau F., Ammar S., Gavard J. (2011). Evaluation of iron oxide nanoparticle biocompatibility. Int. J. Nanomed..

[20] Raynal I., Prigent P., Peyramaure S., Najid A., Rebuzzi C., Corot C. (2004). Macrophage endocytosis of superparamagnetic iron oxide nanoparticles: Mechanisms and comparison of ferumoxides and ferumoxtran-10. Investig. Radiol..

[21] Tate J.A., Petryk A.A., Giustini A.J., Hoopes P.J. (2011). *In vivo*biodistribution of iron oxide nanoparticles: An overview. Proc. SPIE.

[22] Gao Z., Lukyanov A.N., Torchilin V.P. (2002). Diacyllipid-polymer micelles as nanocarriers for poorly soluble anticancer drugs. Nano Lett..

[23] Cinteza L.O., Ohulchanskyy T.Y., Sahoo Y., Bergey E.J., Pandey R.K., Prasad P.N. (2006). Diacyllipid micelle-based nanocarrier for magnetically guided delivery of drugs in photodynamic therapy. Mol. Pharm..

[24] Singh N., Jenkins G.J.S., Asadi R., Doak S.H. (2010). Potential toxicity of superparamagnetic iron oxide nanoparticles (SPION). Nano Rev..

[25] Moore A., Marecos E., Bogdanov A., Weissleder R. (2000). Tumoral distribution of long-circulating dextran-coated iron oxide nanoparticles in a rodent model. Radiology.

[26] Li L., Jiang L.-L., Zeng Y., Liu G. (2013). Toxicity of superparamagnetic iron oxide nanoparticles: Research strategies and implications for nanomedicine. Chin. Phys. B.

[27] Patil U.S., Adireddy S., Jaiswal A., Mandava S., Lee B.R., Chrisey D.B. (2015). *In vitro*/*in vivo* toxicity evaluation and quantification of iron oxide nanoparticles. Int. J. Mol. Sci..

[28] Wang Y.X., Hussain S.M., Krestin G.P. (2001). Superparamagnetic iron oxide contrast agents: Physicochemical characteristics and applications in MR imaging. Eur. Radiol..

[29] Park J., Yu M.K., Jeong Y.Y., Kim J.W., Lee K., Phan V.N., Jon S. (2009). Antibiofouling amphiphilic polymer-coated superparamagnetic iron oxide nanoparticles: Synthesis, characterization, and use in cancer imaging *in vivo*. J. Mater. Chem..

[30] Nasongkla N., Bey E., Ren J., Ai H., Khemtong C., Guthi J.S., Chin S.F., Sherry A.D., Boothman D.A., Gao J. (2006). Multifunctional polymeric micelles as cancer-targeted, MRI-ultrasensitive drug delivery systems. Nano Lett..

[31] Ghio A.J., Turi J.L., Yang F., Garrick L.M., Garrick M.D. (2006). Iron homeostasis in the lung. Biol. Res..

[32] Dunford H.B. (2002). Oxidations of iron(II)/(III) by hydrogen peroxide total plasma malondialdehyde with mild derivatization conditions. Clin. Chem..

[33] Briley-Saebo K., Bjornerud A., Grant D., Ahlstrom H., Berg T., Kindberg G.M. (2004). Hepatic cellular distribution and degradation of iron oxide nanoparticles following single intravenous injection in rats: Implications for magnetic resonance imaging. Cell Tissue Res..

[34] Marklund S.L. (1982). Human copper-containing superoxide dismutase of high molecular weight. Proc. Natl. Acad. Sci. USA.

[35] Tkaczyk J., Vízek M. (2007). Oxidative stress in the lung tissue—Sources of reactive oxygen species and antioxidant defence. Prague Med. Rep..

[36] Bargagli E., Olivieri C., Bennett D., Prasse A., Muller-Quernheim J., Rottoli P. (2009). Oxidative stress in the pathogenesis of diffuse lung diseases: A review. Respir. Med..

[37] Shukla S., Jadaun A., Arora V., Sinha R.K., Biyani N., Jain V.K. (2015). *In vitro* toxicity assessment of chitosan oligosaccharides coated iron oxide nanoparticles. Toxicol. Rep..

[38] Düssman H., Kögel D., Rehm M., Prehn H.M. (2003). Mitochondrial membrane permeabilization and superoxide production during apoptosis. J. Biol. Chem..

[39] Karlsson H.L., Cronholm P., Gustafsson J., Moeller L. (2008). Copper oxide nanoparticles are highly toxic: Acomparison between metal oxide nanoparticles and carbon nanotubes. Chem. Res. Toxicol..

[40] Ahamed M., Alhadlaq H.A., Alam J., Khan M.A., Ali D., Alarafi S. (2013). Iron oxide nanoparticle-induced oxidative stress and genotoxicity in human skin epithelial and lung epithelial cell lines. Curr. Pharm. Des..

[41] Yu M., Huang S., Yu K.J., Clyne A.M. (2012). Dextran and Polymer Polyethylene glycol (PEG) coating reduce both 5 and 30 nm iron oxide nanoparticle cytotoxicity in 2D and 3D cell culture. Int. J. Mol. Sci..

[42] Chen A., Lin X., Wang S., Li L., Liu Y., Ye L., Wang G. (2012). Biological evaluation of Fe_3_O_4_-poly(l-lactide)-poly(ethylene glycol)-poly(l-lactide) magnetic microspheres prepared in supercritical CO_2_. Toxicol. Lett..

[43] Kareyeva A.V., Grivennikova V.G., Vinogradov A.D. (2012). Mitochondrial hydrogen peroxide production as determined by the pyridine nucleotide pool and its redox state. Biochem. Biophys. Acta.

[44] Kareyeva A.V., Grivennikova V.G., Cecchini G., Vinogradov A.D. (2011). Molecular identification of the enzyme responsible for the mitochondrial NADH-supported ammonium-dependent hydrogen peroxide production. FEBS Lett..

[45] Popescu I.M., Cinteza L.O., Hermenean A., Dinischiotu A. (2015). *In vivo* exposure of mice spleen to magnetic nanoparticles encapsulated in phospholipid polymeric micelles; an oxidative stress and structural approach. Dig. J. Nanomater. Biostruct..

[46] Zhu M.T., Feng W.Y., Wang B., Wang T.C., Gu Y.Q., Wang M., Wang Y., Ouyang H., Zhao Y.L., Chai Z.F. (2008). Comparative study of pulmonary responses to nano- and submicron-sized ferric oxide in rats. Toxicology.

[47] Park H.S., Kim S.R., Lee Y.C. (2009). Impact of oxidative stress on lung diseases. Respirology.

[48] MacNee W. (2001). Oxidative stress and lung inflammation in airway disease. Eur. J. Pharmacol..

[49] Repine J.E., Bast A., Lankhorst I. (1997). Oxidative stress in chronic obstructive pulmonary disease. Oxidative Stress Study Group. Am. J. Respir. Crit. Care Med..

[50] Gaharwar U.S., Paulraj R. (2015). Iron oxide nanoparticles induced oxidative damage in peripheral blood cells of rat. J. Biomed. Sci. Eng..

[51] Stadtman E.R. (2006). Protein oxidation and aging. Free Radic. Res..

[52] Castegna A., Drake J., Pocernich C., Butterfield D.A., Hensley K., Floyd R.A. (2003). Protein Carbonyl Levels—An Assessment of Protein Oxidation. Methods in Pharmacology and Toxicology: Methods in Biological Oxidative Stress.

[53] Kowaltowski A.J., Vercesi A.E. (1999). Mitochondrial damage induced by conditions of oxidative stress. Free Radic. Biol. Med..

[54] Forkert P.G., Custer E.M., Alpert A.J., Ansari G.A., Reynolds E.S. (1982). Lactate dehydrogenase activity in mouse lung following 1,1-dichloroethylene: Index of airway injury. Exp. Lung Res..

[55] Dwivedi S., Siddiqui M.A., Farshori N.N., Ahamed M., Musarrat J., Al-Khedhairy A.A. (2014). Synthesis, characterization and toxicological evaluation of iron oxide nanoparticles in human lung alveolar epithelial cells. Colloids Surf. B Biointerfaces.

[56] Fattman C.L. (2008). Apoptosis in pulmonary fibrosis: Too much or not enough?. Antioxid. Redox Signal..

[57] Kuwano K. (2008). Involvement of epithelial cell apoptosis in interstitial lung diseases. Intern. Med..

[58] Hsin Y., Chen C., Huang S., Shih T., Lai P., Chueh P.J. (2008). The apoptotic effect of nanosilver is mediated by a ROS- and JNK-dependent mechanism involving the mitochondrial pathway in NIH3T3 cells. Toxicol. Lett..

[59] Ramkumar K.M., Manjula C., GnanaKumar G., Kanjwal M.A., Sekar T.V., Paulmurugan R., Rajaguru P. (2012). Oxidative stress-mediated cytotoxicity and apoptosis induction by TiO_2_ nanofibers in HeLa cells. Eur. J. Pharm. Biopharm..

[60] Ramesh V., Ravichandran P., Copeland C.L., Gopikrishnan R., Biradar S., Goornavar V., Ramesh G.T., Hall J.C. (2011). Magnetite induces oxidative stress and apoptosis in lung epithelial cells. Mol. Cell. Biochem..

[61] Rath P.C., Aggarwal B.B. (1999). TNF-induced signaling in apoptosis. J. Clin. Immunol..

[62] Lenaz G. (2001). The mitochondrial production of reactive oxygen species: Mechanisms and implications in human pathology. IUBMB Life.

[63] Perel P., Roberts I., Sena E., Wheble P., Briscoe C., Sandercock P., Macleod M., Mignini L.E., Jayaram P., Khan K.S. (2007). Comparison of treatment effects between animal experiments and clinical trials: Systematic review. Br. Med. J..

[64] De Jong M., Maina T. (2010). Of mice and humans: Are they the same?—Implications in cancer translational research. J. Nucl. Med..

[65] Sun S., Zeng H.J. (2002). Size-controlled synthesis of magnetite nanoparticles. J. Am. Chem. Soc..

[66] Lowry O.H., Rosebrough N.J., Farr A.L., Randall R.J. (1951). Protein measurement with the Folin-Phenol reagents. J. Biol. Chem..

[67] Borgmann V., Moon T., Laidler K. (1974). Molecular kinetics of beef heart lactate dehydrogenase. Biochemistry.

[68] Aebi H., Bergmayer H.U. (1984). Catalase *in vitro*. Methods of Enzymatic Analysis.

[69] Paoletti F., Aldinucci D., Mocali A., Caparrini A. (1986). A sensitive spectrophotometric method for the determination of superoxide dismutase activity in tissue extracts. Anal. Biochem..

[70] Beutler E. (1984). Glutathione peroxidase. Red Cell Metabolism. A Manual of Biochemical Methods.

[71] Goldberg D.M., Spooner R.J., Bergmayer H.U. (1983). Glutathione reductase. Methods of Enzymatic Analysis.

[72] Dinischiotu A., Stanca L., Gradinaru D., Petrache S.N., Radu M., Serban A., Armstrong D., Bharali D.J. (2013). Chapter 10. Lipid Peroxidation due to*in vitro* and *in vivo* exposure of biological samples to nanoparticles. Oxidative Stress and Nanotechnology: Methods and Protocols, Methods in Molecular Biology.

[73] Witko V., Nguyen A.T., Descamps-Latscha B. (1992). Microtiter plate assay for phagocyte-derived taurine-chloramines. J. Clin. Lab. Anal..

[74] Fields R., Dixon H.B.F. (1971). Micro method for determination of reactive carbonyl groups in proteins and peptides, using 2,4-dinitrophenylhydrazine. Biochem. J..

[75] Riener C., Kada G., Gruber H.J. (2002). Quick measurement of protein sulfhydryls with Ellman’s reagent and with 4,4′-dithiodipyridine. Anal. Bioanal. Chem..

